# Social cognitive predictors of regular dental visits and mouth self-examination behaviors among the elderly population: An application of the health action process approach model

**DOI:** 10.1371/journal.pone.0293843

**Published:** 2023-11-09

**Authors:** Fatemeh Moghaddam, Katayoun Sargeran, Mahdia Gholami, Jamshid Jamali, Ahmadreza Shamshiri

**Affiliations:** 1 Research Center for Caries Prevention, Dentistry Research Institute, Department of Community Oral Health, School of Dentistry, Tehran University of Medical Sciences, Tehran, Iran; 2 Department of Community Oral Health, School of Dentistry, Tehran University of Medical Sciences, Tehran, Iran; 3 Department of Biostatistics, School of Health, Social Determinants of Health Research Center, Mashhad University of Medical Sciences, Mashhad, Iran; Shahid Beheshti University of Medical Sciences School of Dentistry, ISLAMIC REPUBLIC OF IRAN

## Abstract

**Objective:**

The present study aimed to identify the social cognitive predictors of regular dental visits and mouth self-examination behaviors among the elderly population, based on the Health Action Process Approach (HAPA) model.

**Background:**

Regular dental visits and mouth self-examination can prevent oral and dental problems among the elders. Little information is available regarding the social cognitive predictive factors of these two behaviors.

**Materials and methods:**

A cross-sectional study was conducted in 24 municipality centers in Tehran, Iran in 2021. The centers were selected randomly using a multi-stage cluster sampling method and 301 elderly attendants aged 60≥years participated in the study. Data collection was done using a researcher-made questionnaire including demographic characteristics and the HAPA model constructs for two target behaviors. Data were analyzed using the Smart-PLS version 3.3.9 via correlation and PLS-SEM analysis.

**Results:**

The mean age of the participants was 65.3±5.33 years and 79.7% were female. The SEM analysis showed that Action Self-Efficacy [b (SD) = 0.595 (0.065), P< 0.001] and Risk Perception [b (SD) = 0.218 (0.070), P< 0.002] were predictors of Intention for mouth self-examination but only Action Self-Efficacy [b (SD) = 0.651 (0.043), P< 0.001] was predictor of Intention for regular dental visits. Recovery Self-Efficacy and Planning directly contributed to the prediction of Mouth Self-Examination. The relationship between Maintenance Self-Efficacy and both behaviors is mediated by Planning. Also, the mediating role of Planning between Intention and target behaviors was confirmed.

**Conclusion:**

Action self-efficacy predicted the intention for regular dental visits and mouth self-examination behaviors. The relationship between intention and both behaviors was mediated by Planning. Emphasis on improving Action Self-Efficacy and Intention formation will enhance the effectiveness of interventions aiming at promoting the oral health of the elderly population.

## Introduction

As a result of improved life expectancy and reduced mortality, the global population is aging rapidly [1, 2]. The United Nations has projected that the number of elderly people (aged 60+) will double from 600 million in 2020 to 1.2 billion by 2025 reaching two billion by 2050 [3]. According to the last national census in Iran in 2016, people aged 60 years and over comprise about 9.3% of the total population with a rising trend [4]. Ageing increases the risk of non-communicable chronic diseases dramatically and reduces physical and cognitive abilities [2, 5]. This process is usually associated with low individual immunity, poor nutritional status, and adverse socio-economic factors [6].

Oral health, as part of general health, is also affected [7–10]. Oral and dental problems in the elderly included: 1- Dental caries as a consequence of: age-related hypo-function of salivary glands; use of medications with potential side effect of xerostomia; cariogenic diet; exposure of the root surface related to gingival recession; and poor oral hygiene, 2- Periodontal disease as a result of bacterial plaque accumulation with consequent gingivitis and alveolar bone loss, 3- Tooth loss and Edentulism: The endpoint of caries and periodontal disease; and 4- Oral cancer which its prevalence increases with older age. [1, 7, 9]. Oral diseases, especially in the elders, have serious impacts on the individuals and society in terms of pain, suffering, functional impairment, reduction of chewing performance, diet change, weight loss, limitation of social interactions, low self-esteem, and reduced quality of life [9, 11]. In addition, diseases of the oral cavity are very expensive to treat in most countries [12]. Regular dental visits and mouth self-examination by individuals can help in prevention and early diagnosis of oral diseases, such as oral cancers and root caries, which are common in the older adults [13–18]. Oral self-examination is practical for everyone and is a simple, noninvasive method for early detection of oral cancer. People may not be aware of the normal appearance of their mouth and may not identify the abnormal variations. Therefore, health education on oral self-examination is necessary [14].

Elderly populations face numerous barriers, such as lack of access to dentists, low income levels, high treatment costs, lack of dental insurance, and negative attitudes toward the importance of oral health, which can hinder regular visits to oral health providers [19]. If older people are not aware of oral health problems or do not understand that these problems can be prevented or addressed, they may be less willing to seek dental care in light of the aforementioned challenges.

Older people are less likely to have received preventive education early in life when oral hygiene habits can be most effectively established and are therefore more resistant to change in later life [20]. Hence, oral health promotion programs should focus on improving older people’s perception of the importance of oral health by helping them to integrate dental knowledge into their beliefs [21]. Oral health educational approaches for the elderly should be designed according to lifestyles and abilities to enable them to make decisions to improve personal oral hygiene and oral health. [22]. New approaches to oral health education are required to address personal motivations for preventive behavior [23]. However, a lack of self-regulatory skills is associated with unwillingness to change health behaviors. Therefore, motivation alone will not be adequate to change people’s behavior. To overcome this limitation, self-regulatory processes are believed to operate in agreement with motivational processes to ensure Intention formation [24].

For all age groups traditional approaches to individual oral health education based on information giving and expert advice have been shown to be largely unsuccessful [25]. Furthermore, knowledge increase alone rarely leads to sustained behavior change [23–26]. Thus, there is a need for more effective approaches and models that focus on a broader context that determines the behavior patterns. These models explain how individuals change their behavior and describe their readiness for change [27]. Health behavior change refers to motivational as well as volitional processes, such as adopting and maintaining health-promoting behaviors. It also encompasses a variety of social, emotional, and cognitive factors that sometimes are assumed to operate in concert [28]. Also, knowledge of the behavior determinants, their importance, and their direct and indirect effects on each variable are essential for the advancement of theoretical research as well as intervention development.

**Fig 1:**
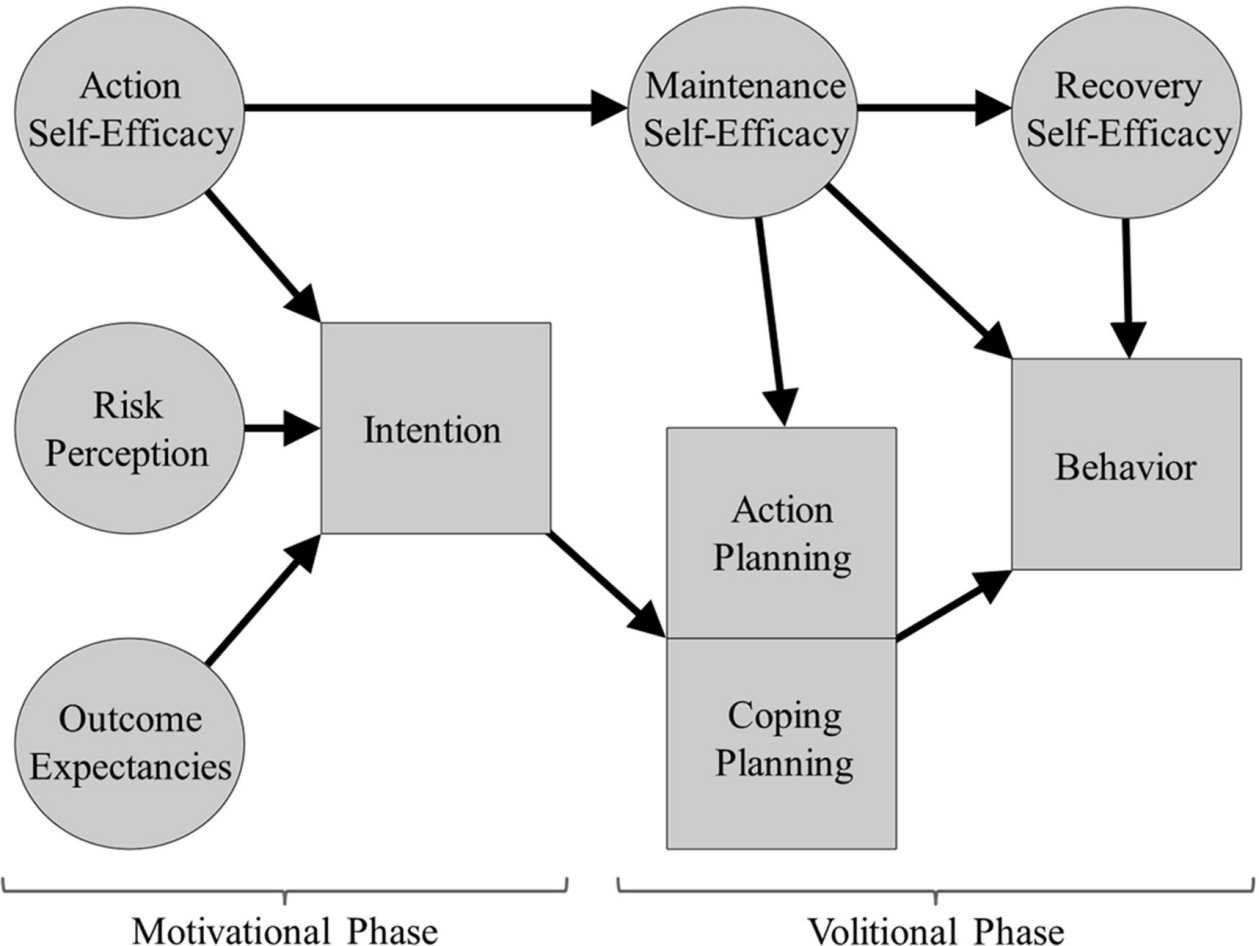
Hypothesized structure of the health action process approach [33].

To establish behavioral change in oral health education, a variety of theories and models have been used [29, 30]. The health action process approach (HAPA) model [31] is a theoretical framework that has been frequently used to understand, explain, and predict several behavioral change mechanisms. The HAPA model is also proposed to bridge the gap between Intention and behavior by providing a variety of beliefs and dispositions that lead to the successful adoption and maintenance of health behaviors [31–33]. The HAPA model considers two different motivational and volitional phases in behavior change (Fig 1):

1- The pre-Intentional phase (motivational), which includes 3 social cognitive constructs: Risk Perception (any negative consequences of the current behavior), Outcome Expectancies (which results to expect from the behavioral change), and Action Self-Efficacy (people assess their belief in themselves to perform the behavioral change) [31, 33] and leads to behavioral Intention.

2- The post-Intentional phase (volitional), which includes Maintenance Self-Efficacy (people assess their own abilities to maintain the new behavior), Action Planning (people make detailed plans on ‘when, where and how’ they will implement the change), Coping Planning (people consider possible barriers and plan how to anticipate these events), Recovery Self-Efficacy (people assess their own abilities to restart the new behavior if a relapse occurs) [34], and target behavior.

Evidence shows that interventional programs based on the HAPA help improve oral health, and the constructs of the HAPA are associated with oral health behaviors [35, 36].

According to a systematic review, in 13 studies the HAPA model was evaluated to assess behavioral changes in oral health. In the majority of the studies, the targeted intervention was flossing and the population consisted of students, adolescents and dental patients [37].

Based on the available data, no study has evaluated the determinants and predictive factors of regular oral health behaviors of the elderly according to the HAPA model. Furthermore, a limited number of studies have used the Partial Least Squares Structural Equation Modeling (PLS-SEM). PLS, a “causal-predictive” advent to SEM, was planned to defeat the obvious dichotomy between explanation and prediction [38]. However, while researchers using PLS-SEM routinely underline the predictive nature of their analyses, model evaluation assessment relies exclusively on metrics designed to assess the path model’s explanatory power [39]. Therefore, the present study aimed to identify social cognitive determinants of regular dental visits and mouth self-examination behaviors among the elderly population, based on the health action process approach (HAPA) model.

## Methods

### Study design and participants

The present analytical, cross-sectional study was conducted on the elderly aged 60 years or more in Tehran, Iran from January to March 2021.

### Sampling frame

Tehran is divided into 22 municipality districts, and each district has several municipality neighborhood houses that operate within the framework of policies and macro-urban management programs in the neighborhood. A municipality neighborhood house is a public, non-profit, and financially self-governing institution that seeks to attract, organize, and promote public participation in cultural, health, and social activities at the neighborhood level. The health center is one of the active parts of the neighborhood house. This study was conducted in health centers for better access to the target group, especially during the Covid-19 pandemic.

### Sampling method and sample size

Multi-stage cluster sampling was used for sampling assuming the health centers of selected neighborhood houses as clusters. Six out of the 22 municipality districts were selected randomly, including districts 1 and 5 from the north, 19 and 21 from the south, and 11 and 13 from the center of Tehran. Finally, 24 municipality neighborhood houses (four selected randomly from each district) were included. From each center, 12–15 eligible elderly subjects were selected using the convenience sampling method.

According to Hair Jr et al. (2014), the minimum sample size for PLS-SEM is determined by the often-cited 10 times rule indicating that the sample size should be equal to the larger of:

a. 10 times the largest number of formative indicators used to measure a single construct, or

b. 10 times the largest number of structural paths directed at a particular construct in the structural model [40].

In the present study according to HAPA model, we had 13 structural paths for each of the two study behaviors (Fig 1). Therefore, the minimum sample size should be considered 130*2 = 260. Because of model complexity and number of samples available, it can safely be concluded that a sample size of 301 was acceptable for this study.

#### Inclusion and exclusion criteria

The inclusion criteria were age 60 years or older, living in the selected districts, being a member of the health center, being able to communicate with research facilitators, and completing the informed consent form. Elderly people with uncontrolled systemic diseases that led to person’s inability to communicate or cooperate with the researchers and non-Iranian citizens were excluded.

### Study questionnaire and variables

An instrument was designed to measure the study variables, including three parts:

1) Socio-demographic characteristics

The first part evaluated socio-demographic characteristics including age, gender, income, education, employment status, living status, and medical history.

2) Oral health behaviors

The second part evaluated two oral health behavior variables using the following questions:

- Last dental visit (When was the last time you visit a dentist?). The answer options were as follows: within the past 6 months, within the past 6–12 months, between 1 and 2 years ago, between 2 and 5 years ago, more than 5 years ago, and never. Then, the options were dichotomized as follows: a score of 2 was given to less than 12 months ago and a score of 1 was given to more than 12 months.
- Mouth self-examination (Do you check your mouth every night after brushing/ cleaning the denture?). A score of 1 was given to “Yes” and a score of 0 was assigned to “No” [14].

The sociodemographic data and two oral health behaviors were self-reported by the participants.

3) Health Action Process Approach questionnaire

The third part of questionnaire was designed for oral health behaviors based on the HAPA constructs. The validity and reliability of the questionnaire were assessed before data collection.

The HAPA questionnaire included the following constructs and questions. Each construct was measured with a single item (question) for each behavior and the answers were rated on a 7-point Likert scale from 1 to 7. The scale was based on the Schwarzer’s recommendations [41–43].

a) Risk Perception (RP): The questions were as follows:

- “If I do not check and examine my mouth regularly, I don’t notice lesions and they may extend asymptomatically.”
- “If I do not visit the dentist at least once a year, my oral and dental problems will not be detected in time.”

The answers were rated on a 7-point Likert scale from 1 (very unlikely) to 7 (very likely).

b) Outcome Expectancies (OE): The questions were as follows:

- “If I examine my mouth regularly, I will identify any lesions in early stages.”
- “If I visit the dentist at least once a year, less money will be spent on my teeth.”

The answers were rated on a 7-point Likert scale from 1 (strongly disagree) to 7 (strongly agree).

c) Action Self-Efficacy (A SE): The questions were as follows:

- “I’m sure I can check and examine my mouth every day after brushing or cleaning dentures, even if I’m tired.”
- “I’m sure I can visit the dentist at least once a year, even if I have a problem with time.”

The answers were rated on a 7-point Likert scale from true (1) to definitely true (7).

d) Intention: The questions were as follows:

- “I plan to check and examine my mouth every day after brushing or cleaning dentures in the coming weeks or months.”
- “I plan to have annual visits to the dentist for examination or dental treatment.”

The answers were rated on a 7-point Likert scale from 1 (strongly disagree) to 7 (strongly agree).

e) Action Planning (AP): The questions were as follows:

- “I have a precise plan to check and examine my mouth every day after brushing or cleaning my dentures.”
- “I have a precise plan to visit the dentist at least once a year for examination of oral cavity and teeth.”

The answers were rated on a 7-point Likert scale from 1 (strongly disagree) to 7 (strongly agree).

f) Coping Planning (CP): The questions were as follows:

- “I already have concrete plans. If I fail to examine my oral cavity, I will do it later on the same day or the next morning.”
- “I already have concrete plans. If I am too busy to visit the dentist, I will do it later the next week or the next month.”

The answers were rated on a 7-point Likert scale from 1 (strongly disagree) to 7 (strongly agree).

g) Maintenance Self-Efficacy (M SE): The questions were as follows:

- “I’m sure I can check and examine my mouth every day after brushing or cleaning dentures even if it takes a long time to become part of my daily routine.”
- “I’m sure I can visit the dentist at least once a year even if access is difficult in terms of place and time.”

The answers were rated on a 7-point Likert scale from not at all true (1) to definitely true (7).

h) Recovery Self-Efficacy (R SE): The sample questions were as follows:

-” I’m sure I can start checking and examining my mouth again regularly after brushing or cleaning my denture even if I have not examined for a month.”
- “I’m sure I can schedule to visit the dentist again regularly even if I postpone my plans several times.”

The answers were rated on a 7-point Likert scale from not at all true (1) to definitely true (7).

### Validity and reliability of the questionnaire

The initial set of items was based on the definitions of the HAPA constructs and instructions on how to create HAPA-related scale [24, 32, 33, 41–43]. After the initial item generation and refinement by the primary research team, the validity and reliability of the questionnaire were assessed. The content validity index (CVI) and content validity ratio (CVR) were calculated for each item [44, 45]. These two indices were applied to evaluate the essentiality, relevance, clarity, and simplicity of the questions by 9 experts (8 community oral health specialists and 1 health education and promotion specialist). All of the items had an acceptable CVR (≥0.77) and CVI (≥0.88). For face validity, the questionnaire was completed by 10 elderly subjects and their comments were used to make minor changes in the questionnaire.

The reliability of the instrument was tested using intra-class correlation coefficient (ICC). The questionnaire was completed twice over a period of one week by 30 elderly people aged 60 years and older in a pilot study and the ICC was calculated for each construct. The ICC was above 0.70 in test–retest analysis and had good agreement [46].

### Data collection

The questionnaires were filled out through face-to-face interviews. For every participant, the questions were read one by one followed by the optional answers. If necessary, the interviewer repeated the items to ensure that the participant understood them well. The response rate was almost 100%; hence, this study was performed with complete data and finally, the data of 301 elderly people were analyzed.

### Data analysis

In this study, descriptive data are presented as frequency, mean, and standard deviation. Partial Least Squares Structural Equation Modeling (PLS-SEM) was used to test the relationship between the latent variables, including Risk Perception, Outcome Expectancies, self-efficacy, Intention, planning, and the outcome variables i.e. oral health behaviors (regular dental visits and mouth self-examination).

Bootstrapping is a non-parametric method that allows testing path coefficients in PLS-SEM. Analysis of the inner model quality includes an assessment of the coefficient of determination (R^2^), path coefficient, and effect size (F^2^). Coefficient of determination is used to test the explanatory and predictive power of the structural model. The R^2^ values range from 0 to 1, with a value closer to one indicating a better model. R^2^ values of 0.19, 0.33, and 0.67, as suggested by Urbach and Ahlemann [47], correspond to small, medium, and large explanatory powers, respectively. F^2^ values of 0.02, 0.15, and 0.35, as suggested by Cohen [48], correspond to weak, moderate, and strong effects. Analysis of the path coefficient can describe the association between variables in a hypothesis. The path coefficient values range from −1 to +1, with a coefficient closer to 1 indicating a stronger association, whether the association is positive or negative.

The SEM can analyze the direct and indirect relationships between study variables (HAPA constructs). Indirect relationship: If HAPA construct contributed to the prediction of a behavior via mediating construct and Direct relationship: If HAPA construct contributed to the prediction of a behavior without mediating construct.

All data were analyzed using SPSS 25.0 and Smart PLS version 3.3.9 software and the test significance was a two-tailed alpha <0.05, but for bivariate correlations, significances was considered at the 0.01 level (2-tailed).

### Ethics statement

This study was approved by the Research Ethics Committee of Tehran University of Medical Sciences (IR.TUMS.DENTISTRY.REC.1399.102). Before completing the questionnaires, the researcher explained the study purpose and obtained written informed consent from the elderly for voluntary participation. All information collected from respondents during this research will be kept confidential. All identifiable details of the participants will be separated from the coded details. The identifiable details and data entered on the computer will be password-protected and only accessible to the researchers.

## Results

### Participants characteristics

Participants sociodemographic characteristics and frequencies of two oral health behaviors (regular dental visits and daily mouth self-examination) are shown in Table 1. The average age of participants was 65.31 years (SD = 5.33), most participants were female (79.7%, n = 240), and 17.6% lived alone. Only 36.5% of the participants have had a dental visit during the last year, and 54.8% check the mouth soft tissue every day after brushing or cleaning dentures.

**Table 1:** Participants characteristics and oral health behaviors (n = 301).

**Variable**	**N**	**%**
**Age** (Mean± SD)	65.3±5.33	
**Gender**		
Female	240	79.7
Male	61	20.3
**Education Level**		
Illiterate	40	13.3
Primary school	108	35.9
Diploma	108	35.9
Bachelor degree	36	12
Master/Ph.D. degree or above	9	3
**Employment status**		
In-service staff	49	16.3
Retiree	72	23.9
Unemployed	8	2.7
Housewife	172	57.1
**Monthly household income ($)**		
Below 165	98	32.6
166–332	107	35.5
333–499	75	24.9
500–749	14	4.7
750 or more	7	2.3
**Living status**		
With spouse only	107	35.5
With family	127	42.2
Alone	53	17.6
other	14	4.7
**Last dental visit**		
During the last 12 months	110	36.5
More than 12 months	191	63.5
**Mouth self-examination**		
Yes	165	54.8
No	136	45.2

### Characteristics of latent constructs

The mean, standard deviation, and bivariate correlation between HAPA constructs and dental visit behavior were shown in Table 2. Action Planning and Coping Planning are strongly correlated with regular dental visits behavior.

**Table 2:** Descriptive analysis for target behavior regular dental visit: Means, standard deviations, and bivariate correlations.

Variable	M	SD	1	2	3	4	5	6	7	8	9
1. Outcome Expectancies	4.65	1.52	1	0.737**	0.487**	0.471**	0.346**	0.341**	0.361**	0.458**	0.214**
2. Risk Perception	4.94	1.43		1	0.560**	0.516**	0.423**	0.418**	0.417**	0.534**	0.263**
3. Action Self-Efficacy	4.08	1.61			1	0.743**	0.781**	0.775**	0.835**	0.765**	0.467**
4. Intention	3.79	1.89				1	0.624**	0.612**	0.626**	0.740**	0.434**
5. Action Planning	3.71	1.45					1	0.981**	0.903**	0.739**	0.801**
6. Coping Planning	3.68	1.45						1	0.908**	0.726**	0.802**
7. Maintenance Self-Efficacy	3.93	1.47							1	0.767**	0.723**
8. Recovery Self-Efficacy	4.07	1.42								1	0.596**
9. Dental Visits	1.37	0.48									1

** Correlation is significant at the 0.01 level (2-tailed). SD: Standard Deviation, M: Mean

The mean, standard deviation, and bivariate correlation between HAPA constructs and mouth self-examination behavior were shown in Table 3. As displayed in Table 3, Action Self-Efficacy has a strong correlation with mouth self-examination behavior.

**Table 3:** Descriptive analysis for target behavior mouth self-examination: Mean, standard deviations, and bivariate correlations.

Variable	M	SD	1	2	3	4	5	6	7	8	9
1. Outcome Expectancies	4.43	1.46	1	0.863**	0.707**	0.684**	0.673**	0.639**	0.633**	0.687**	0.557**
2. Risk Perception	4.72	1.33		1	0.756**	0.732**	0.696**	0.691**	0.675**	0.732**	0.567**
3. Action Self-Efficacy	4.44	1.31			1	0.813**	0.776**	0.809**	0.800**	0.802**	0.797**
4. Intention	4.41	1.40				1	0.749**	0.833**	0.776**	0.807**	0.681**
5. Action Planning	3.97	1.34					1	0.749**	0.747**	0.769**	0.635**
6. Coping Planning	4.21	1.38						1	0.830**	0.810**	0.675**
7. Maintenance Self-Efficacy	4.33	1.37							1	0.852**	0.647**
8. Recovery Self-Efficacy	4.55	1.30								1	0.676**
9. Mouth Self-Examination	0.55	0.50									1

**. Correlation is significant at the 0.01 level (2-tailed). SD: Standard Deviation, M: Mean

### Structural model

**Fig 2:**
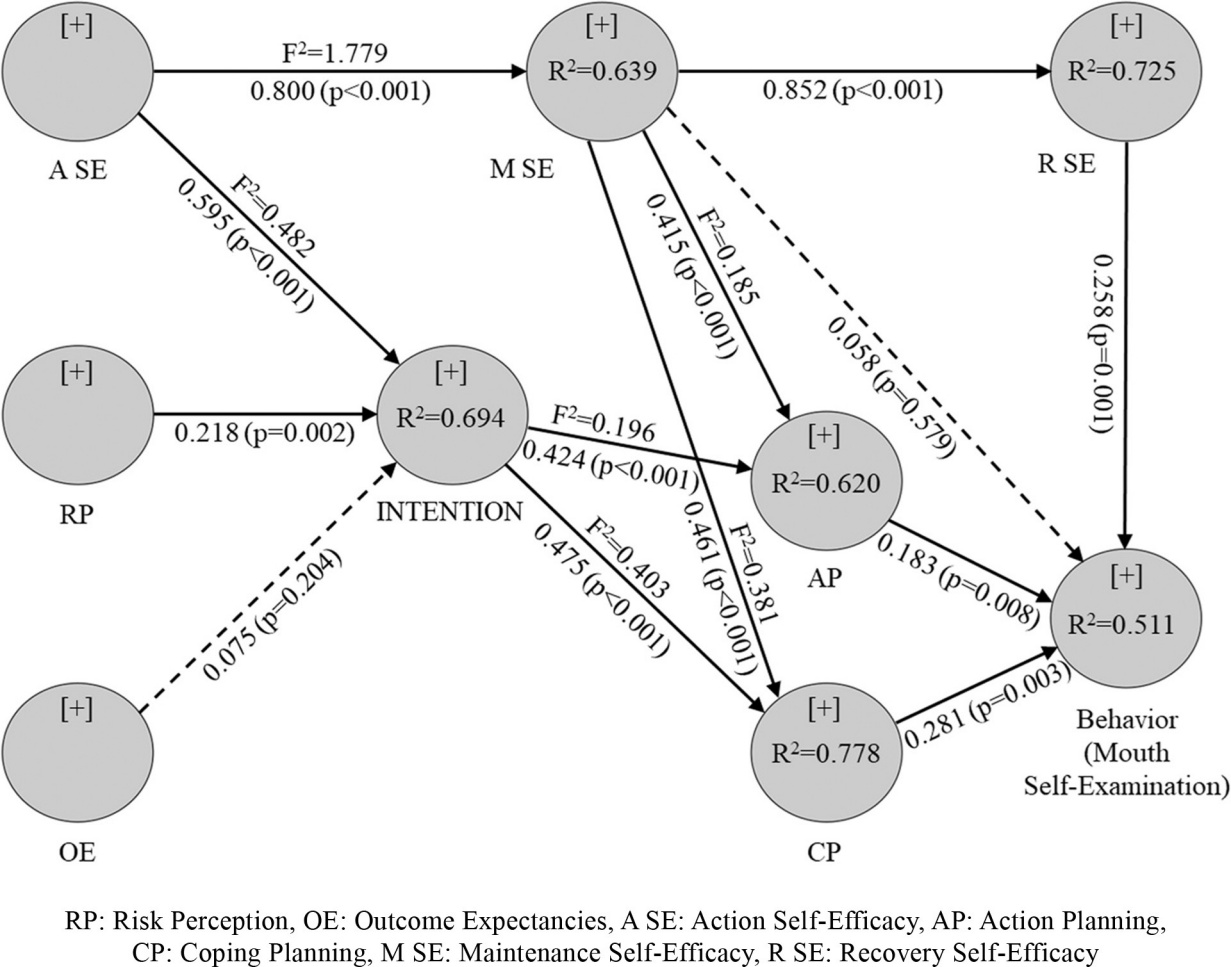
SEM analysis results of the HAPA model for mouth self-examination.

**Fig 3:**
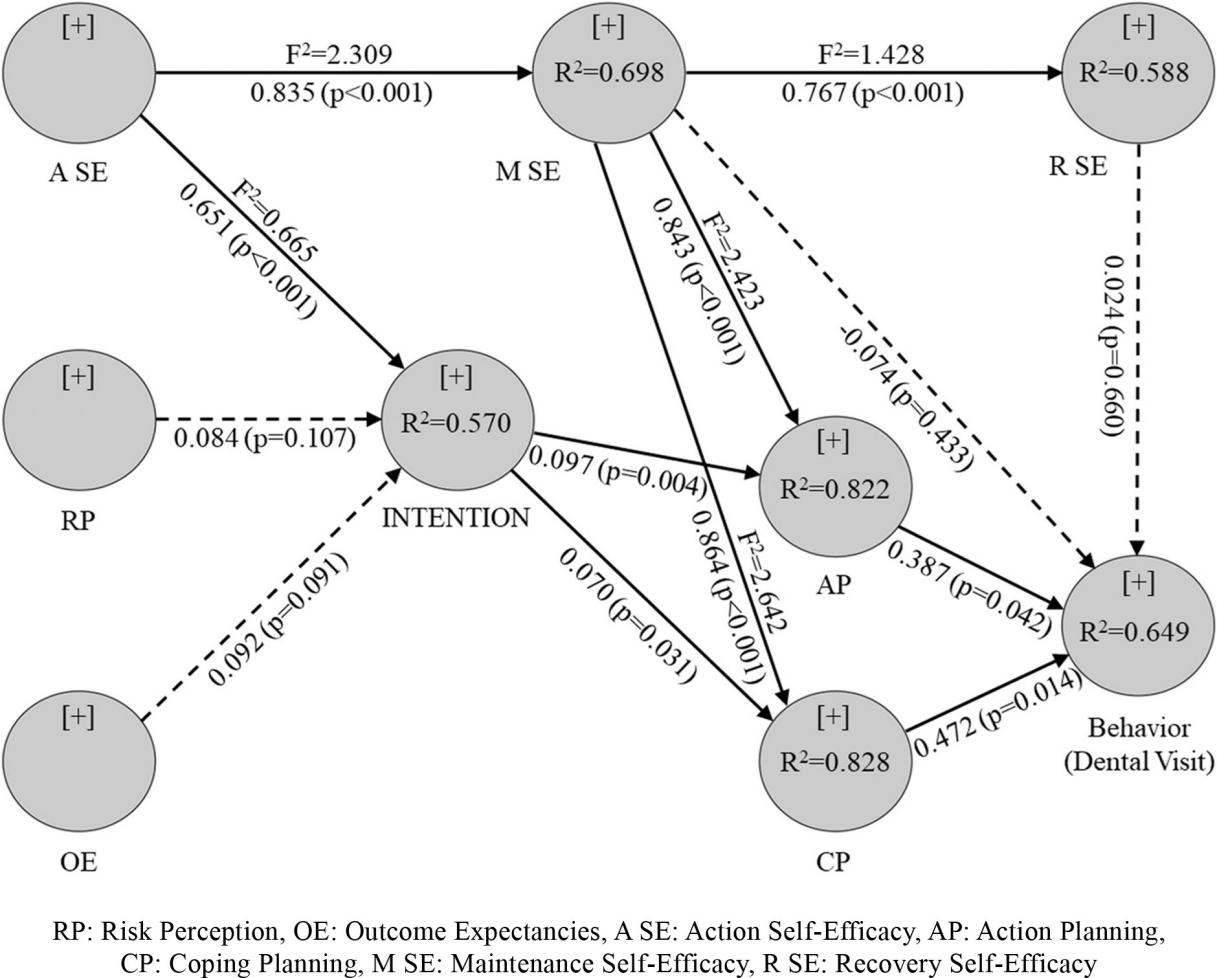
SEM analysis results of the HAPA model for regular dental visits.

The path analysis of latent variables in the HAPA model includes an assessment of the coefficient of determination (R^2^), path coefficient (b), and effect size (F^2^). Structural model results for mouth self-examination were shown in Table 4 and Fig 2 and for regular dental visits behavior were illustrated in Table 5 and Fig 3.

**Table 4:** SEM analysis results of mouth self-examination behavior.

Relationship	Coefficient	SD	T	p
AP → Behavior: Mouth Self-Examination	0.183	0.069	2.656	0.008
CP → Behavior: Mouth Self-Examination	0.281	0.095	2.953	0.003
Intention → AP	0.427	0.067	6.370	<0.001
Intention → CP	0.475	0.088	5.412	<0.001
M SE → AP	0.415	0.068	6.112	<0.001
M SE → Behavior: Mouth Self-Examination	0.058	0.104	0.556	0.6
M SE → CP	0.461	0.094	4.923	<0.001
M SE → R SE	0.852	0.019	45.226	<0.001
OE → Intention	0.075	0.059	1.273	0.2
R SE → Behavior: Mouth Self-Examination	0.258	0.078	3.295	0.001
RP → Intention	0.218	0.070	3.131	0.002
A SE → Intention	0.595	0.065	9.189	<0.001
A SE → M SE	0.800	0.023	34.997	<0.001

OE: Outcome Expectancies, RP: Risk Perceptions, A SE: Action Self Efficacy, AP: Action Planning, CP: Coping Planning, M SE: Maintenance Self Efficacy, R SE: Recovery Self Efficacy

**Table 5:** SEM analysis results of regular dental visits behavior.

Relationship	Coefficient	SD	T	p
AP → Behavior: Dental Visit	0.387	0.185	2.088	0.04
CP → Behavior: Dental Visit	0.472	0.183	2.583	0.01
Intention → AP	0.097	0.033	2.898	0.004
Intention → CP	0.070	0.031	2.236	0.03
M SE → AP	0.843	0.030	28.424	<0.001
M SE → Behavior: Dental Visit	-0.074	0.098	0.757	0.5
M SE → CP	0.864	0.026	33.215	<0.001
M SE → R SE	0.767	0.032	23.670	<0.001
OE → Intention	0.092	0.056	1.648	0.1
R SE → Behavior: Dental Visit	0.024	0.053	0.451	0.7
RP → Intention	0.084	0.052	1.629	0.1
A SE → Intention	0.651	0.043	15.150	<0.001
A SE → M SE	0.835	0.027	30.599	<0.001

OE: Outcome Expectancies, RP: Risk Perceptions, A SE: Action Self Efficacy, AP: Action Planning, CP: Coping Planning, M SE: Maintenance Self Efficacy, R SE: Recovery Self Efficacy

#### Mouth self-examination behavior

For the behavioral Intention of mouth self-examination, Action Self-Efficacy [b (SD) = 0.595 (0.065), P< 0.001] and Risk Perception [b (SD) = 0.218 (0.070), P< 0.002] were positively related to behavioral Intention. That is, the greater the Action Self-Efficacy and Risk Perception, the better the Intention. Intention was positively associated with action and Coping Planning [b (SD) = 0.427 (0.067), P< 0.001], [b (SD) = 0.475 (0.088), P< 0.001]. Also, Maintenance Self-Efficacy was positively associated with action and Coping Planning [b (SD) = 0.415 (0.068), P< 0.001], [b (SD) = 0.461 (0.0947), P< 0.001]. Action Planning and Coping Planning were positively correlated with mouth self-examination behavior [b (SD) = 0.183 (0.069), P< 0.008] [b (SD) = 0.281 (0.95), P< 0.003] (Table 4). So that higher scores on Action and Coping Planning were associated with higher scores of mouth self-examination behavior.

Fig 2 shows the determination coefficient (R^2^) or strength of the predictive model created for mouth self-examination behavior, Intention, planning, Maintenance Self-Efficacy, and Recovery Self-Efficacy. The model could explain the variations in 51.1% of behavior, 69.4% of Intention, and 69.8% of planning (sum of action and Coping Planning). The medium effect size (F^2^) was found for maintenance to Action Planning and Intention to planning. A large effect size was found for self-efficacy to Intention and Maintenance Self-Efficacy, Intention to Coping Planning, and Maintenance Self-Efficacy to Coping Planning.

#### Dental visit behavior

For the behavioral Intention of regular dental visit, Action Self-Efficacy [b (SD) = 0.651 (0.043), P< 0.001] was positively related to behavioral Intention. Intention was positively associated with Action and Coping Planning [b (SD) = 0.097 (0.033), P< 0.004], [b (SD) = 0.070 (0.031), P< 0.026]. Also, Maintenance Self-Efficacy was positively associated with action and Coping Planning [b (SD) = 0.843 (0.030), P< 0.001], [b (SD) = 0.864 (0.026), P< 0.001]. Action Planning and Coping Planning were positively correlated with regular dental visits [b (SD) = 0.387(0.185), P< 0.037] [b (SD) = 0.472 (0.183), P< 0.010]. (Table 5). So that higher scores on Action and Coping Planning were associated with higher scores on regular dental visits behavior.

Fig 3 shows the determination coefficient (R^2^) or strength of the predictive model created for regular dental visits behavior, Intention, planning, Maintenance Self-Efficacy, and Recovery Self-Efficacy. The model could explain the variations in 64.9% of behavior, 57% of Intention, and 82.5% of planning (sum of action and Coping Planning). A large effect size (F^2^) was found for self-efficacy to Intention and Maintenance Self-Efficacy, and Maintenance Self-Efficacy to Coping Planning, Action Planning, and Recovery Self-Efficacy.

### Mediation analysis

A mediator effects analysis revealed that Risk Perception can positively correlate with mouth self-examination [b (SD) = 0.046 (0.019), P< 0.01] indirectly through behavioral Intention and planning. So that better mouth self-examination behavior was associated with increased Risk Perception, Intention and Planning scores. Moreover, further analysis also found that Action Self-Efficacy can be positively correlated with regular dental visits [b (SD) = 0.613 (0.027), P< 0.001] and mouth self-examination [b (SD) = 0.512 (0.037), P< 0.001] indirectly through Intention and Planning as the mediators. Mediation analysis findings are shown in Table 6.

**Table 6:** Mediating effect.

Path	Direct effect		Indirect effect		Total effect	
	coefficient	p	coefficient	p	coefficient	p
RP → Intention → Planning* → Mouth Self-Examination	-	-	0.046	0.01	0.046	0.01
A SE → Intention → Planning → Mouth Self-Examination	-	-	0.512	<0.001	0.512	<0.001
A SE → Intention → Planning → Dental Visit	-	-	0.613	<0.001	0.613	<0.001

RP: Risk Perceptions, A SE: Action Self Efficacy

* Planning: Sum of Action Planning and Coping Planning

## Discussion

Due to the theoretical strengths of the HAPA model, it is widely used in many fields, such as measuring social distancing behavior [49], physical activity [50–52], dieting [53], and oral hygiene [24, 54, 55]. The present study was conducted to examine the determinants and predictive factors of regular dental visits and mouth self-examination in the elderly. The results confirmed that the HAPA model could explain and predict the initiation and maintenance of behaviors of interest (regular mouth self-examination and dental visits) in the elderly population. The model included the initiation (motivational) and maintenance (volitional) stages of regular mouth self-examination and dental visits behaviors. In the initiation of mouth self-examination behavior, the results of the integrated model showed that Action Self-Efficacy and Risk Perception were predictors of Intention. These two constructs were indirectly related to mouth self-examination behavior through behavioral Intention and planning. However, outcome expectations did not significantly predict Intention. As for behavior maintenance, the results showed that Action Planning, Coping Planning, and Recovery Self-Efficacy were directly related to mouth self-examination behavior. Maintenance Self-Efficacy had an indirect relationship with mouth self-examination through Action Planning, Coping Planning, and Recovery Self-Efficacy. However, Action Planning and Coping Planning had moderating effects on the relationship between Intention and mouth self-examination.

In the initiation of dental visits behavior, the results of the integrated model showed that Action Self-Efficacy was predictor of Intention. Also, only Action Self-Efficacy had an indirect relationship with dental visits behavior through behavioral Intention and planning. As for behavior maintenance, the results showed that Action Planning, Coping Planning, and Recovery Self-Efficacy were directly related to dental visits behavior.

Maintenance Self-Efficacy had an indirect relationship with dental visits through Action Planning, Coping Planning, and Recovery Self-Efficacy. However, Action Planning and Coping Planning had moderate effects on the relationship between Intention and dental visits.

A significant finding of this study was the moderator role of self-efficacy. This finding affords precise support for the prominence of phase-specific multiple types of self-efficacy in the HAPA model [52]. Action Self-Efficacy operates on the left side of the model (motivational phase or initiation of HAPA model) as a first-stage moderator, whereas Maintenance Self-Efficacy operates on the right side (volitional phase or maintenance of HAPA model) as a second-stage moderator. The participants with higher levels of self-efficacy reported higher levels of planning. In addition, self-efficacy predicted the desired health behaviors, i.e., dental visits and mouth self-examination, in the elderly.

These findings are consistent with studies that indicated Action Self-Efficacy as a predictor of dental flossing and stronger self-efficacy was related to improved oral health behavior, which in turn was associated with better oral health status [54, 56, 57]. Moreover, it has been demonstrated that an increase in self-efficacy may be an important component of interventions designed for changing oral health behavior [24, 37, 58, 59].

Outcome Expectancies did not significantly predict Intention for both behaviors. There may be some reasons. The first reason may be a low knowledge level of oral disease and the importance of oral health in general health. The second reason may be incorrect beliefs about oral diseases; for example, one may think that tooth loss and other oral diseases that occur with age are natural and unpreventable processes. Furthermore, the elderly may be concerned about the high costs of dental treatments while they may not be aware that if their disease is detected in the early stages, the treatment costs may be much lower. It is considerable that the planning factor (action and Coping Planning) is a mediator variable for Intention to behavior and Maintenance Self-Efficacy to outcomes pathways [60].

The findings of the present study support the tenants of the HAPA in that the effects of Intention on behavior are mediated via planning [33, 41, 51]. Xu et al. also found that Maintenance Self-Efficacy indirectly predicted physical activity through planning [61]. Paxton showed that the initiation and behavior maintenance required Action Planning and Coping Planning [62].

Self-regulatory skills such as planning are required to enable individuals to overcome barriers that may potentially hinder their intentions to accomplish behaviors [63]. This suggests that Action and Coping Planning are important mediators of the maintenance of mouth self-examination and dental visit behaviors in the elderly. Therefore, practical interventions should be designed to rise the elders’ willingness to improve planning and to increase their ability confront difficulties or barriers in performing oral health behaviors to promote behavior maintenance [60].

The skills of the planner and the quality of the plan are important for the positive effect of the planning on the intended behavior. Effective planning may follow the SMART principles. Plans need to be specific (a narrow behavior), measurable, assignable (who will perform), realistic, and time-related (when to perform the action). These are well-known principles that help to guide individuals in behavior planning (55).

Moreover, a large body of evidence argues that planning predicts various health behaviors. Both Action Planning and Coping Planning contributed to the prediction of dental visits and mouth self-examination. Finally, it should be noted that while the model fit the data relatively well, there may be other models that also fit the data. An alternative model that includes direct paths from Action Self-Efficacy to behavior, Intention to behavior, and Recovery Self-Efficacy to action/Coping Planning warrants future consideration.

In the present study, 17.6% of the participants lived alone. This is near to the prevalence of 18.1% reported from our country [64]. The prevalence of living alone in old ages varies worldwide, and has increased recently due to population aging. A global comparative study showed that the prevalence of older women living alone ranged from 45–50% to 5–10% and among men, varied from 25% to below 5%. In most countries, living alone is more frequent in women due to the higher life expectancy [64].

More than 63% of the study participants did not have a dental visit for more than 12 months. Regular dental visit is a key element in prevention and early detection of oral diseases. It is important to find out the reasons for the patients not seeking routine dental check-ups, in order to develop effective interventions to improve the oral health of the population. High costs of dental treatments, low income [65], and dental anxiety [66] are the most common reasons for infrequent dental visits.

Almost 50% of our study participants reported that they check their mouth every day. The possible reason for this high percentage is that participants may tend to choose a socially desirable answer, when completing the questionnaire. Furthermore, they were obviously not aware of mouth self-examination method and its steps, because they did not engage in any educational program in this regard and just had a quick glance at the oral soft tissue.

### Strengths, limitations, and future directions

Municipality neighborhood houses are important settings offering an appropriate opportunity to reach elderly population and community members. The present multi-center study was conducted in municipality neighborhood houses of different geographical and socioeconomic levels in Tehran, making the sample more representative of the elderly population, especially elderly women, in the capital city.

This study had some limitations. First, all variables were self-reported measures, which may cause recall bias and measurement error. Second, most of the study participants were women. This fact was in compliance with the pattern of the attendance to health centers, especially in working hours. Men are more likely to have a job after official retirement and are busy in working times of a day. However, this should be deliberated in the future studies. Third, demographic characteristics were not included as co-variables in the model. It is suggested that the influence of socio-demographic characteristics, especially gender be considered in forthcoming studies. Furthermore, due to the cross-sectional nature of the study design, causal inferences can not be made and all mediation effects should be interpreted with caution. A next step to verify the causal role of the social cognitive predictors of regular dental visits and mouth self-examination behaviors among the elderly population is to examine them in interventional trials.

## Conclusion

The HAPA model showed a good applicability in the prediction of two oral health behaviors in a sample of elderly subjects aged ≥60 years old. Action Self-Efficacy and Risk Perception were predictors of Intention for mouth self-examination but only Action Self-Efficacy was the predictor of Intention for regular dental visits. Recovery Self-Efficacy and Planning directly contributed to the prediction of Mouth Self-Examination. The relationship between Maintenance Self-Efficacy and both behaviors is mediated by Planning. Also, the mediating role of Planning between Intention and target behaviors was confirmed.

Therefore, dental team and advocates of geriatric oral health should place more emphasis on increasing multiple types of self-efficacy specially action and maintenance self-efficacy and Intention formation to select interventions directed at improving oral health care-seeking behavior.

## Supporting information

S1 Dataset (XLSX)
